# Dacryoadenitis associated with linear scleroderma *en coup de sabre*: A case report and review of literature

**DOI:** 10.1002/ski2.391

**Published:** 2024-04-22

**Authors:** Eva Oustabassidis, Shay Keren, Joel David, Jonathan H. Norris

**Affiliations:** ^1^ Oxford Eye Hospital John Radcliffe Hospital Oxford UK; ^2^ Rheumatology Department Nuffield Orthopaedic Centre Oxford UK

## Abstract

Dacryoadenitis in the setting of linear scleroderma *en coup de sabre* (LScs) is an association that has not previously been described in the scientific literature. The purpose of this case report is to describe the co‐existence of LScs and chronic dacryoadenitis and how it was managed. We report the case of a 42‐year‐old woman who presented with a 4‐month history of left upper eyelid swelling with radiological enlargement of the left lacrimal gland on orbital CT and MRI imaging. Clinical examination revealed a left erythematous, swollen upper eyelid with lateral conjunctival injection and a palpable left lacrimal gland. An ipsilateral band‐like cutaneous depression in the fronto‐temporal region was also noted, which extended to the ipsilateral upper eyelid and brow. Serology revealed nucleolar antinuclear antibodies. A further incisional biopsy of the lacrimal gland confirmed chronic inflammatory changes and fibrous tissue. Based on both the histological and clinical findings, a diagnosis of dacryoadenitis in association with LScs was made. Oral methotrexate was commenced. The patient responded well with less frequent episodes of eyelid swelling and reduced periocular pain. This case describes for the first time, the ophthalmological manifestation of chronic dacryoadenitis in association with linear scleroderma *en coup de sabre*.

## Graphical abstract

Dacryoadenitis in the setting of linear scleroderma en coup de sabre (LScs) is an association that has not previously been described in the scientific literature. The purpose of this case report is to describe the co‐existence of LScs and chronic dacryoadenitis and how it was managed. Methotrexate can effectively and safely manage both conditions and should be considered as an early therapy to avoid exposing patients to the detrimental side‐effects of steroids.

## 1 INTRODUCTION

Linear scleroderma, a subtype of localized scleroderma, is a condition of unknown origin^1^ in which an area of skin and subcutaneous tissue and occasionally deeper tissue become thickened and scarred with increased collagen deposition. Linear scleroderma *en coup de sabre* (LScs) is a subset of linear scleroderma and is characterized by a scar‐like band of skin predominantly affecting the scalp and upper face.^2^ Several ophthalmological findings in scleroderma have been previously reported.^3^ Fibrotic eyelid skin changes leading to thickening, tightness, telangiectasia, blepharophimosis or lagophthalmos have been reported. Other ocular manifestations include keratoconjunctivitis sicca, episcleritis, uveitis, and dacryocystitis. We present a unique case of chronic dacryoadenitis associated with linear scleroderma *en coup de sabre* which to our knowledge has not been reported. Written consent from patient was obtained for publication. This case report follows the CARE Guidelines.^4^

## 2 NARRATIVE

**FIGURE 1:**
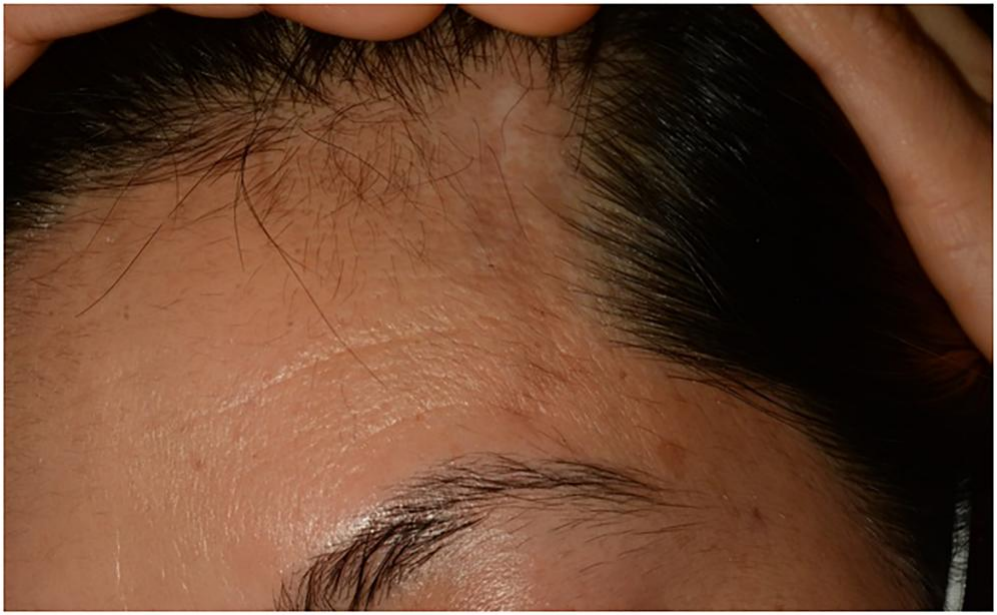
Left‐sided fronto‐temporal band‐like skin changes suggestive of linear scleroderma ‘en coup de sabre’.

A 42‐year‐old healthy female presented to the Oculoplastic clinic with a 4‐month history of left upper eyelid swelling associated with ipsilateral conjunctival redness. History revealed investigation in relation to a history of fever, joint pain, head rash, and a left forehead scar that followed an episode of right lateral upper eyelid swelling. She described left periocular pain extending to the ipsilateral forehead. On examination, visual acuity was 6/6 in both eyes and ocular movements were full with normal optic nerve function. On the left side, there was a lateral upper eyelid oedema associated with lateral bulbar conjunctival injection. The left lacrimal gland was palpable and enlarged. A linear depression of the skin was noted to be extending from the lateral left upper eyelid, through the lateral eyebrow to the fronto‐temporal hairline. The skin changes were suggestive of an *en coup de sabre* seen in linear scleroderma (Figure 1).

**FIGURE 2:**
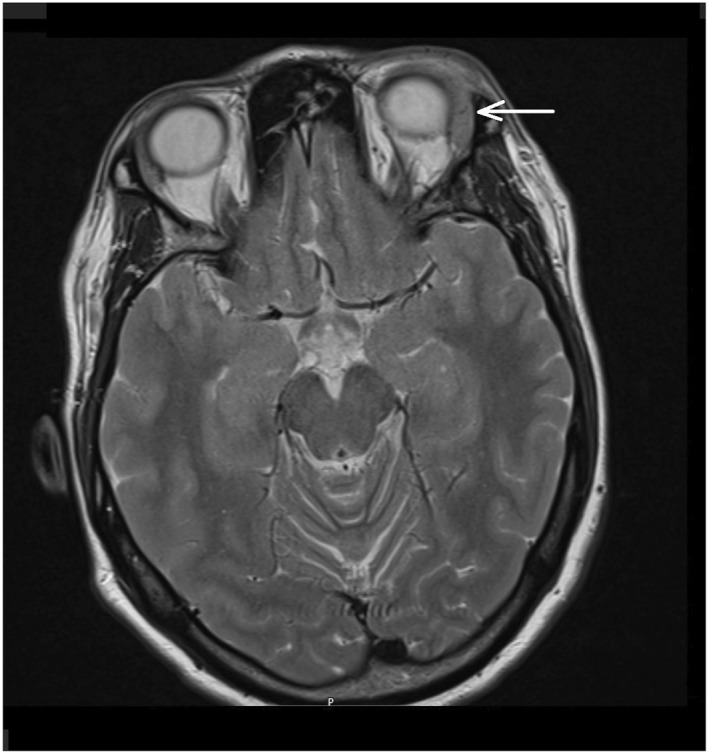
MRI T2‐weighted, axial plane. Enlargement of left lacrimal gland.

Blood work showed normal full blood count, CRP, ESR, and ACE. Immunoglobulin blood levels, IgG4 subclass, as well as serum protein electrophoresis were normal. ENA, ANCA, and dsDNA antibodies were negative. ANA was positive with a titre of 320 (laboratory standards 0–80) and a nucleolar pattern on immunofluorescence. Orbital CT and MRI demonstrated an enlarged left lacrimal gland (Figure 2).

Initial management by the referring hospital included oral Co‐amoxiclav and IV methylprednisolone 500 mg for 3 days followed by oral prednisone on a tapering regimen which improved eyelid inflammation. Any dosage of prednisolone below 30 mg resulted in recurrence of symptoms.

**FIGURE 3:**
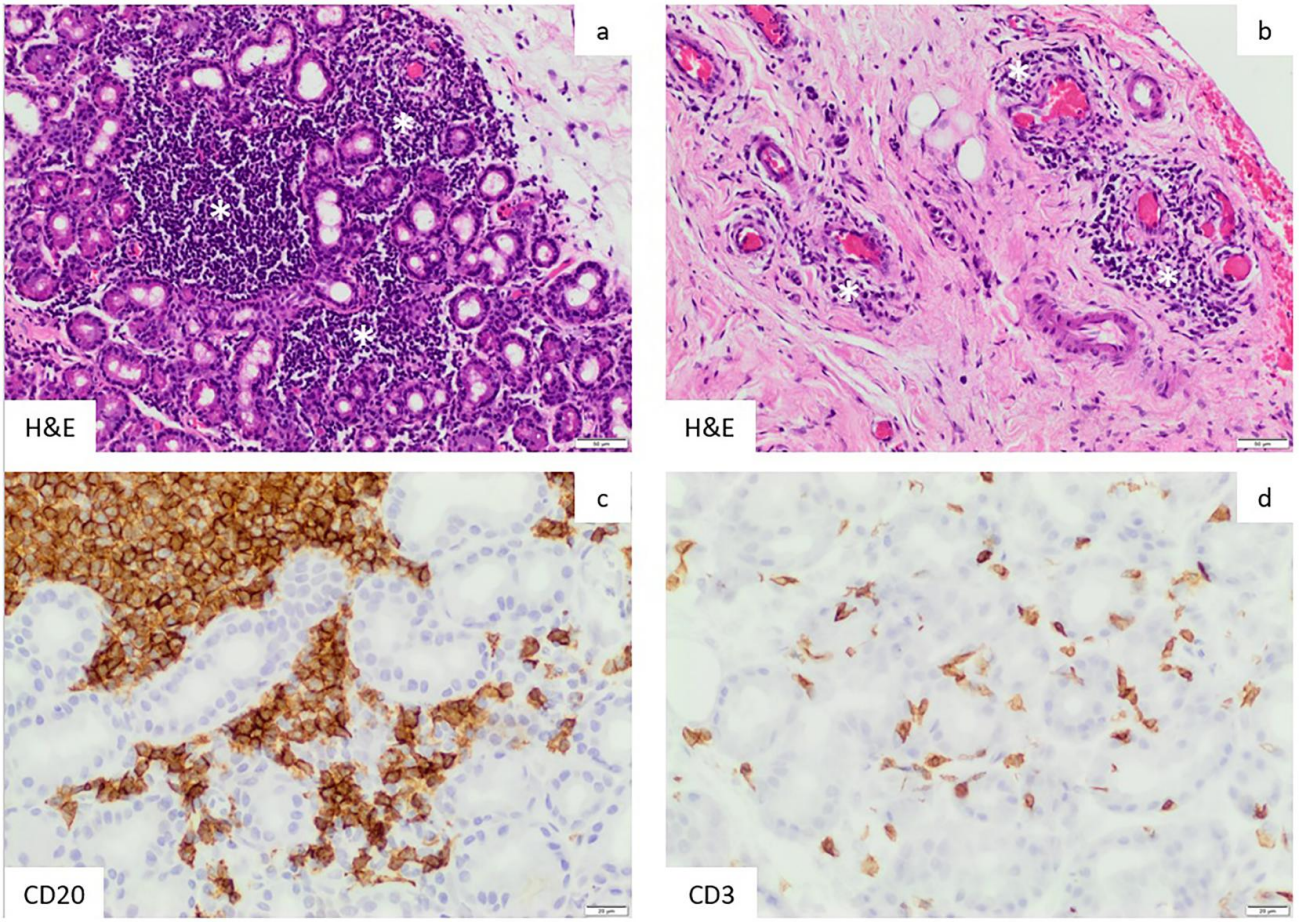
Histopathology. Biopsy tissue demonstrates lymphocytic dacryoadenitis (a, c, d) with the inflammation extending into adjacent fibrotic soft tissues in a perivascular pattern (b). White asterisks in (a) and (b) indicate collections of lymphocytes. The brown reaction product in (c) and (d) highlights individual B‐cells (CD20+) and T‐cells (CD3+), respectively.

A left lacrimal gland biopsy via an anterior orbitotomy was performed under general anaesthesia. Oral prednisolone was progressively reduced to 10 mg a day prior to surgery to reduce the steroid masking effect of histopathological signs. Histopathology (Figure 3) revealed a patchy chronic lymphoplasmacytic inflammation of the lacrimal gland with no lymphoid cell atypia or granuloma. The infiltrate comprised a mixture of CD20 positive B‐cells and CD3 positive T‐lymphocytes. There was no evidence of IgG4 expression. The inflammation extended into adjacent fibrous tissue and orbital muscle with a perivascular pattern; there was no vasculitis.

In the first year following the lacrimal biopsy, the patient remained relatively asymptomatic, bar persistent left upper eyelid oedema without tenderness. At 12 months, however, she developed a left‐sided sectoral episcleritis in conjunction with eyelid swelling and left‐sided headaches and so was commenced on oral methotrexate (MTX) 15 mg once per week for 2 weeks then increased to 20 mg once per week with folic acid 5 mg per week. The patient was reviewed 3 months later and reported a sustained reduction in the left periocular pain as well as less frequent episodes of eyelid swelling. There was no evidence of episcleritis or dacryoadenitis. The *en coup de sabre* scar showed no sign of active inflammation. She was maintained on MTX 20 mg per week and the steroid successfully weened.

## 3 PATIENT PERSPECTIVE

Before the diagnosis, I used to have a constant pain to my left eye with headaches. I needed to regularly take ibuprofen and omeprazole which upset my stomach. I felt relieved when doctors found out what was the underlying issue and also why I had this scar on my forehead. Thankfully, once I started taking MTX, my headache and my eyelid swelling reduced and I was able to enjoy my activities again.

## 4 DISCUSSION

Localised scleroderma is a non‐hereditary, autoimmune condition of unknown aetiology. The pathogenesis is still not fully understood. Immune activation is thought to be the initial step in the pathological process and is targeted against the endothelium of small blood vessels.^5^ This process results in tissue fibrosis and vascular structural changes such as destructive vasculopathy and proliferative obliterative vasculopathy. Similar perivascular changes and fibrosis were also seen in the lacrimal gland specimen in this case. The area of dermal inflammation and subsequent fibrosis leads to palpable thickening and hyperpigmentation.^6^ Changes also affect the deeper layers of the subdermis, fat, fascia, muscle, and can lead to bone damage and atrophy of the tongue and jaw deformity.^7^

Scleroderma *en coup de sabre* refers to ‘the blow of a sword’ and is a subset of localised scleroderma usually manifesting as a unilateral linear atrophic depressed cutaneous band with possible sclerosis localised on the forehead or scalp. It affects children more commonly and is more predominant in females than males (3:1).^6^

Ocular and orbital manifestations in linear scleroderma observed so far are poorly described in the literature. Reported findings include atrophy of the eyelid, eyelashes and eyebrow which may be associated with enophthalmos. These manifestations are thought to result from late trophic changes in the overlying skin sclerosis.^8^ Ipsilateral episcleritis, such as in our patient, anterior uveitis, and extraocular muscles involvement (thinning and fibrosis) may occasionally occur. Refractive changes and pseudopapilloedema have also been reported in the paediatric community.^9^ Dry eye is a less well reported association with LScs.^10^ One report describes tear secretion deficiency related to scleroderma without knowing the exact pathological mechanism.^11^ It may be that this was related to low‐grade dacryoadenitis.

One paper describes LScs with associated nasolacrimal duct obstruction and prolonged dacryocystitis^12^ occurring as a result of occlusive fibrosis. The finding of dacryoadenitis in our case report suggests deep organ involvement in the context of LScs and identifies an ophthalmological association of the disease which has not previously been described in literature. Dacryoadenitis refers to the inflammation of the lacrimal gland which is located supero‐temporally to the globe. Clinical manifestation is with a painful red enlarged lacrimal gland associated with an S‐shape deformity of the upper eyelid. Biopsy of the lacrimal gland is key if an underlying immune disease is suspected. Inflammatory causes may respond to steroids or demonstrate a chronic relapsing course requiring long‐term immunomodulatory therapy to maintain remission.^13^

Methotrexate has been extensively studied for the treatment of LScs and 100% responding rate has been reported.^14^

## 5 CONCLUSION

This case illustrates for the first time the finding of chronic dacryoadenitis in the context of LScs. Recognising the association and investigation with slit lamp examination, imaging, and histopathology is key to enhance optimum care of patients and reduces the risk to develop long‐term ocular dryness form scarred lacrimal gland which could lead to corneal infection and ulceration. Methotrexate can effectively and safely manage both conditions and should be considered as an early therapy to avoid exposing patients to the detrimental side‐effects of steroids.

## AUTHOR CONTRIBUTIONS

**Eva Oustabassidis**: Conceptualization (lead); writing – original draft (lead); writing – review & editing (lead). **Shay Keren**: Writing – review & editing (supporting). **Joel David**: Writing – review & editing (supporting). **Jonathan H. Norris**: Supervision (lead); writing – review & editing (supporting).

## CONFLICT OF INTEREST STATEMENT

None to declare.

## ETHICS STATEMENT

Not applicable.

## Data Availability

Data sharing is not applicable to this article as no new data were created or analyzed in this study.
